# Innovative Fibrous Materials Loaded with 5-Nitro-8-hydroxyquinoline via Electrospinning/Electrospraying Demonstrate Antioxidant, Antimicrobial and Anticancer Activities

**DOI:** 10.3390/antiox12061243

**Published:** 2023-06-09

**Authors:** Mariya Spasova, Nikoleta Stoyanova, Nasko Nachev, Milena Ignatova, Nevena Manolova, Iliya Rashkov, Ani Georgieva, Reneta Toshkova, Nadya Markova

**Affiliations:** 1 Laboratory of Bioactive Polymers, Institute of Polymers, Bulgarian Academy of Sciences, Akad. G. Bonchev St, bl. 103A, BG-1113 Sofia, Bulgaria; nstoyanova@polymer.bas.bg (N.S.); nachev_n@polymer.bas.bg (N.N.); manolova@polymer.bas.bg (N.M.); rashkov@polymer.bas.bg (I.R.); 2 Institute of Experimental Morphology, Pathology and Anthropology with Museum, Bulgarian Academy of Sciences, Akad. G. Bonchev St, bl. 25, BG-1113 Sofia, Bulgaria; ageorgieva@bas.bg (A.G.); rtoshkova@bas.bg (R.T.); 3 Institute of Microbiology, Bulgarian Academy of Sciences, Akad. G. Bonchev St, bl. 26, BG-1113 Sofia, Bulgaria; markn@bas.bg

**Keywords:** 5-nitro-8-hydroxyquinoline, electrospinning, electrospraying, cellulose acetate, water-soluble polymer, antioxidant, antimicrobial, anticancer activities

## Abstract

A new type of fibrous mat based on a cellulose derivative—cellulose acetate (CA) or CA and water-soluble polymers (polyvinylpyrrolidone, PVP or poly(vinyl alcohol), PVA)—loaded with the model drug 5-nitro-8-hydroxyquinoline (5N) was fabricated via electrospinning or electrospinning in conjunction with electrospraying. Scanning electron microscopy (SEM), X-ray diffraction analysis (XRD), Fourier-transform infrared spectroscopy (FTIR), water contact angle measurements and ultraviolet-visible spectroscopy (UV-Vis) were used for the complex characterization of the obtained novel material. The decoration of CA fibers with a water-soluble polymer containing the drug resulted in the facilitation of wetting and fast drug release. The 5N-containing fibrous material showed antioxidant activity. Moreover, the proposed materials’ antibacterial and antifungal properties were tested against *S. aureus*, *E. coli*, *P. aeruginosa* and *C. albicans*. Well-distinguished, sterile zones with diameters above 3.5 cm were observed around all 5N-containing mats. The mats’ cytotoxicity toward HeLa carcinoma cells and normal mouse BALB/c 3T3 fibroblasts was assessed. The 5N-*in*-CA, PVP,5N-*on*-(5N-*in*-CA) and PVA,5N-*on*-(5N-*in*-CA) fibrous mats possessed anticancer efficacies and much lower levels of toxicity against normal cells. Therefore, the as-created novel electrospun materials, which are based on polymers loaded with the drug 5N via electrospinning/electrospraying, can potentially be applied for topical wound healing and for local cancer therapy.

## 1. Introduction

Electrospinning is a highly versatile and attractive technique for the preparation of micro- and nanosized materials via the application of an external electric field to a polymer melt or solution [1,2]. As a result of their excellent characteristics, including a large specific surface area, high porosity and 3D structural design, the materials obtained via electrospinning are potential applicants for the pharmaceutical, medicinal, cosmetic and agricultural fields [1,2,3,4,5]. One-pot electrospinning and simultaneous electrospinning and electrospraying offer opportunities for combining polymers with various low-molar-mass, biologically active compounds and for the preparation of biologically active materials of diverse designs. The specific properties associated with their nano-size enable the sustained release of drugs, leading to a reduction in the cytotoxic effects of drugs and an improvement in their therapeutic effects [6,7].

Cellulose acetate (CA) can be easily derived at a low cost from cellulose, the most abundant natural polymer. CA is of interest as a drug carrier because it possesses a set of fascinating properties: non-toxicity, biodegradability, biocompatibility, recyclability, chemical persistence, thermal constancy and relatively good mechanical and barrier properties [8,9,10,11]. In addition, CA can be easily electrospun [8,12,13]. Polyvinylpyrrolidone (PVP) and poly(vinyl alcohol) (PVA) were selected for use in the fibrous materials utilized for biomedical purposes because these nonionogenic, water-soluble polymers are biocompatible and possess very low levels of toxicity. It has been shown that PVP- or PVA-based electrospun materials can serve as suitable carriers of poorly water-soluble drugs because PVP or PVA increase their rate of dissolution in aqueous solutions [14,15,16]. Previously, some of us have reported that when PVP was present in fibers, it facilitated the release of the sparingly water-soluble drug curcumin from polylactide- and CA-based electrospun fibrous materials [17,18]. Drug-loaded fibrous materials with incorporated PVP or PVA have been shown have prospects for use in biomedical applications [19,20,21,22]. Such systems have been reported to exert good antioxidant, antimicrobial, anticancer and/or wound-healing properties.

8-Hydroxyquinoline and its derivatives possess a wide variety of biological properties: anticancer, antibacterial, antifungal, antioxidant, antiviral and anti-inflammatory properties [23,24]. Their low levels of toxicity to humans are another of their characteristics [25]. Previously, we obtained electrospun fibrous materials based on synthetic [26,27] and natural polymers [28,29,30,31] with incorporated 8-hydroxyquinoline derivatives. These materials displayed strong antimicrobial and/or anticancer activities. We have also shown the preparation of antibacterial fibrous materials via the covalent attachment of an 8-hydroxyquinoline derivative (5-amino-8-hydroxyquinoline) to mats electrospun from styrene–maleic anhydride copolymers [32]. Recently, some of us have successfully fabricated electrospun fibrous materials made from chitosan derivatives, polyetheramines, polylactid and 8-hydroxyquinoline-derivatives and their complexes with copper and iron ions [33,34]. We found that these fibrous mats exhibit good anticancer and/or antimicrobial activities. As far as we know, the literature contains no data for the fabrication of electrospun mats from CA, PVP (or PVA) and 5-nitro-8-hydroxyquinoline (5N). The original contribution of this study is the use of the beneficial features of the polysaccharide derivative (CA), water-soluble synthetic polymers (PVP and PVA) and a drug, 5N, which possesses diverse biological activities, via electrospinning or electrospinning in conjunction with electrospraying. The strategy used herein enables the prospective application of the fabricated materials as wound dressings and for local cancer treatment.

The purpose of this contribution is to investigate the potential of fabricating novel 5N-loaded fibrous materials based on CA or CA and PVP (or PVA) with various designs, using one-pot electrospinning or a conjunction of electrospinning and electrospraying. We fully studied the fibrous morphology and its chemical and structural composition as well as wetting. A disk diffusion assay was used to evaluate the antibacterial and antifungal activities of the created novel materials toward *S. aureus*, *E. coli*, *P. aeruginosa* and *C. albicans*. The in vitro release profile of 5N from the novel materials was investigated as well. Both the antioxidant activities and the in vitro anticancer efficacies of the novel materials were examined in relation to the composition of the polymer matrix.

## 2. Materials and Methods

### 2.1. Materials

Cellulose acetate (CA, Sigma-Aldrich, St. Louis, MO, USA) with ${\bar{M}}_{n}$ 30,000 g/mol and DS 39.8%, polyvinylpyrrolidone K25 (PVP, Fluka, Buchs, Switzerland) with Mr ~ 24,000 g/mol, poly(vinyl alcohol) (PVA, Sigma-Aldrich, St. Louis, MO, USA) with Mw 31,000–50,000 g/mol, 98–99% hydrolyzed, and 5-nitro-8-hydroxyquinoline (5N, Sigma-Aldrich, St. Louis, MO, USA) were used. Furthermore, the following materials were delivered from Sigma-Aldrich, Darmstadt, Germany: acetone, Dulbecco Modified Eagle’s Medium (DMEM), 3-(4,5-Dimethylthiazol-2-yl)-2,5-diphenyltetrazolium bromide (MTT), ethidium bromide (EtBr), acridine orange (AO) and 4′,6-diamidino-2-phenylindole (DAPI). Moreover, FlowLab, Australia, supplied the trypsin–EDTA and orange. Scientific, Braine-l’Alleud, Belgium supplied the disposable consumables. *S. aureus* strain 749, *E. coli* strain 3588, *P. aeruginosa* strain 1390 and *C. albicans* strain 74 were supplied from the National Bank for Industrial Microorganisms and Cell Cultures (NBIMCCs), Bulgaria. The growth medium used for the cultivation of the bacteria was a peptic digest of animal tissue; beef extract with final pH of 6.5 was purchased from Fluka. The American Type Cultures Collection (ATCC, Rockville, MD, USA) supplied the HeLa human cervical cancer cells (ATCC, CCL-2) and mouse BALB/3T3 clone A31 cells (ATCC CCL-163). All the chemicals were of analytical grade and were used as received without any further purification.

### 2.2. Preparation of Fibrous Materials via Electrospinning/Electrospraying

#### 2.2.1. Preparation of CA and 5N-in-CA Mats

Fibrous CA materials were obtained via electrospinning a CA solution in acetone/water 80/20 *v*/*v* at a polymer concentration of 10 wt%. The 5N-in*-*CA fibers were prepared by mixing a CA/5N solution in acetone/water 80/20 *v*/*v* in which the CA concentration was 10 wt%. The 5N concentration was 10 wt% with respect to the polymer weight. The thus-prepared CA and CA/5N solutions were subjected to electrospinning. The electrospinning equipment contained a custom-made high voltage power supply, an infusion pump (NE-300 Just Infusion^TM^ Syringe Pump (New Era Pump Systems Inc., Farmingdale, NY, USA)) for constant rate of delivery for the spinning solution, a syringe that ended with a positively charged metal needle and a grounded rotating collector made of aluminum. The electrospinning process was performed at a constant voltage of 25 kV, the feeding rate of the spinning solution was 3 mL/h, the tip-to-collector distance was 20 cm, the collector rotation speed was 1000 rpm, the room temperature was 22 ° C and the relative humidity was 51%. The fabricated materials were then additionally dried under reduced pressure at 25 °C for 8 h.

#### 2.2.2. Preparation of PVP,5N-on-(5N-in-CA) and PVA,5N-on-(5N-in-CA) Mats via Electrospinning in Conjunction with Electrospraying

The 5N-*in-*CA fibers decorated with PVP or PVA nanoparticles containing 5N were noted as PVP,5N-*on*-(5N-*in*-CA) and PVA,5N-*on-*(5N-*in*-CA) mats, respectively. These types of fibrous materials were created via simultaneous electrospinning and electrospraying techniques. A CA spinning solution (polymer concentration: 10 wt% in acetone_80_/water_20_) containing 5N at a concentration of 10 wt% with respect to the polymer weight was prepared and used for electrospinning. Simultaneous electrospraying was performed using diluted mixed spinning solutions of PVP (concentration: 2 wt%) or PVA (concentration: 2 wt%) with 5N (concentration: 10 wt% with respect to the polymer weight) in a mixture of solvent water/ethanol, 70/30 *v*/*v*. In this case, the PVP or PVA served as a sticking agent to adhere the 5N onto the 5N-*in*-CA fibers during electrospraying. For the electrospinning in conjunction with electrospraying, two separate syringes were used. They were placed in two infusion pumps on both sides of the collector. The angle between the pumps was 180°. The CA,5N solutions were supplied at a speed of 3.0 mL per h. The feeding rate of the PVP,5N or PVA,5N solutions were 2.0 mL per h. The distance from the tip to the collector was 20 cm when CA,5N electrospinning was performed and 15 cm when electrospraying the PVP,5N or PVA,5N solutions. The voltage of 25 kV, generated using a common high-voltage power supply, was applied to the needles of the two syringes. The grounded drum in which the electropun/electrosprayed materials were collected was rotated with a speed of 1000 rpm. The fabricated samples were placed under reduced pressure at 25 °C to remove solvent residues. The amount of the 5N loaded in the PVP,5N-*on*-(5N-*in*-CA) and PVA,5N-*on*-(5N-*in*-CA) mats was 10 wt% with respect to the total polymer weight (determined spectrophotometrically (λ_max_ 351 nm, acetone)).

### 2.3. Characterization Methods

The morphological analysis of the fabricated fibrous materials was carried out using a Jeol JSM-5510 (JEOL Co., Ltd., Tokyo, Japan) scanning electron microscope. Prior to the SEM examination, the fibrous samples were vacuum-coated with gold. ImageJ software was used to determine the mean fiber diameter of the fibers and particles by manually measuring at least 50 randomly selected fibers [35].

The 5N-loaded mats were examined using fluorescence microscopy (Leika DM 5000B, Wetzlar, Germany) to show that 5N was present in the fibers or in particles on the fiber surfaces. A fluorescence filter cube I3 (bandpass filter: 450–490 nm) was mounted in the fluorescence microscope’s turret.

Attenuated total reflection Fourier transform infrared (ATR-FTIR) spectroscopy was carried out in order to characterize the fabricated fibrous materials. An IRAffinity-1 spectrophotometer produced by Shimadzu Co., Kyoto, Japan, was used to capture the FTIR spectra. The spectra were recorded from 4000 to 600 cm^−1^.

The Easy Drop shape analysis system DSA 10-MK2, produced by Krüss, Hamburg, Germany, was used to calculate the values of the static contact angles. A computer-controlled dosing system deposited a water drop (10 μL) on the surfaces of the fibers. Ten measurements for each sample were performed.

The obtained fibrous materials were subjected to XRD analysis to examine their crystalline structures. A D8 Bruker Advance powder diffractometer, produced by Bruker, Billerica, MA, USA, and equipped with a filtered CuK radiation source and a luminous detector (step of 0.02° and counting time of 1 s/step), was used to record the XRD patterns.

The 5N content loaded in the mats was measured using a DU 800 UV spectrophotometer delivered by Beckman Coulter, Brea, CA, USA, after dissolving the samples in acetone. The actual amount of 5N was measured via UV–vis spectrophotometry at a wavelength of 351 nm. The experiments were repeated three times.

The in vitro 5N release profiles were studied at 37 °C using an acetate buffer solution with a constant ionic strength of 0.1 (CH_3_COONa/CH_3_COOH) at a pH of 5.5. The samples (10 mg) were immersed in a JULABO SW23 shaking water bath with a controlled temperature which was produced in Allentown, PA, USA, and were stirred with a 100 mL buffer solution. Aliquots of the test solution were withdrawn at specific time intervals, and the amount of released 5N was determined using a DU 800 UV–Vis spectrophotometer (Beckman Coulter, Brea, CA, USA) at a wavelength of 445 nm. The withdrawn volumes were replaced with fresh buffer solution. Calibration curves (correlation coefficient R = 0.999) were used to determine the released drug. The 5N release behavior was investigated in triplicate, and all data were averaged.

The antioxidant activities of free 5N and the prepared fibrous materials were assessed using a radical scavenging test with 2,2-diphenyl-1-picrylhydrazyl (DPPH). DPPH (a stable purple color radical), which reacts with an antioxidant to form a light yellow 2,2-diphenyl-1-picryl-hydrazyl-hydrate, which is the reduced product that can be detected using a spectrophotometer. Ascorbic acid (vitamin C) is a strong reducing agent frequently used to clean free radicals. Ethanol solutions of the 5N (5 × 10^−3^ M) or CA, 5N-*in-*CA, PVP,5N-*on*-(5N-*in*-CA) and PVA,5N-*on-*(5N-*in*-CA) mats were immersed in a solution of DPPH in ethanol with concentration of 1 × 10^−4^ M. Vitamin C was used as a positive control. Each solution was placed in the dark for half an hour. The solutions’ absorbances at 517 nm were determined using a DU 800 UV-Vis spectrophotometer (Beckman Coulter, Brea, CA, USA) to evaluate their antioxidant activities (AA%):

$$\mathrm{Inhibition},\;\mathrm{AA},\%=\left[\frac{\left(A_{\mathrm{DPPH}}-A_{\mathrm{sample}}\right)}{A_{\mathrm{DPPH}}}\right]\times \;100\;$$

where the A_sample_-DPPH• is the solution absorption at 517 nm after the addition of the 5N solution or fibrous materials, and A_DPPH•_—is the absorption for DPPH• solution at 517 nm. Each experiment was repeated three times.

### 2.4. Antibacterial and Antifungal Assessments

The antibacterial and antifungal activities of the fabricated fibrous materials placed in contact with pathogenic microorganisms were assessed. For these purposes, the Gram-positive bacteria *S. aureus*, the Gram-negative bacteria *E. coli* and *P. aeruginosa* and the fungi *C. albicans* were used. For the antibacterial and antifungal tests, discs with diameters of 17 mm were cut. The solid agar nutrient medium used contained 4% glucose, 2% pectin, 0.1% yeast extract and 1.2% Sabouraud agar. The medium surface was inoculated with a suspension of 24 h cell cultures of *S. aureus*, *E. coli*, *P. aeruginosa* or *C. albicans* (with concentrations of 5 × 10^5^ cells mL^−1^). Then, on the surface of the agar, one disc of the fibrous mat was placed onto each dish. The Petri dishes with bacteria were stored at 37 °C for 24 h, and the fungi dishes were stored for 48 h. Then, the mean inhibition zone diameters formed around the samples were determined using Image J software by measuring at least 10 measurements in 10 different directions for each zone.

### 2.5. Cytotoxicity Assessment by MTT Cell Viability Assay

An MTT viability test was used to evaluate the influence of the prepared samples on HeLa cells and normal fibroblasts [36]. DMEM with 10% FBS, 100 U/mL penicillin and 0.1 mg/mL streptomycin and 5% CO_2_ in a CO_2_ incubator at 37 °C were used for the incubation of cultures. When the cells reached 90% confluence, they were trypsinized and counted using a hemocytometer. The concentration of cells was 1 × 10^5^ cells per well. The fibrous materials were sterilized with UV light (30 min), and the sterilized mats were then placed in contact with HeLa and mouse fibroblasts for 24 and 48 h at 37 °C in a humidified environment containing 5% CO_2_. The cell lines were treated with free 5N as well. The cells were cultured with the samples, and then they were incubated for 3 h at 37 °C in the MTT solution. Then the supernatants were removed. Next, a lysing solution (100 μL) (DMSO/ethanol 1:1) was poured into each well. An ELISA plate reader produced by TECAN, SunriseTM, Grodig/Salzburg, Austria, was used to evaluate the MTT assay. The following equation was used to determine the cell viability:

cell viability (%) = OD_570_ (experimental)/OD_570_ (control) × 100

### 2.6. Dual Staining with AO and EtBr for Studying Apoptotic Induction

*N*,*N*,*N*′,*N*′-tetramethylacridine-3,6-diamine (AO) and 3,8-diamino-5-ethyl-6-phenylphenanthridin-5-ium bromide (EtBr) were applied to assess the morphological alterations of the HeLa tumor and mouse fibroblast cells. In order to form a monolayer, the cells (concentrations of 2 × 10^5^ cells × mL^−1^) were plated on glass lamellas, placed at the bottom of 24-well plates and incubated for 24 h at 37 °C in a CO_2_ incubator. After that, the electrospun materials were sterilized by UV light and placed into 24-well plates for 24 h. Then, the samples were removed, and the glass lamellas were washed twice with phosphate-buffered saline (PBS, pH 7.4) to eliminate unattached cells. The lamellas were stained with AO and EtBr at a ratio of 1:1 (10 μg/mL) and immediately observed using a fluorescence microscope (Leika DM 5000B, Wetzlar, Germany).

### 2.7. DAPI Staining Assay

The morphologies of the cell nuclei were observed via staining with 4′,6-diamidino-2-phenylindole (DAPI), according to the manufacturer’s protocol. The cells with concentrations of 1 × 10^5^ cells/well were cultivated with samples on coverslips in tissue culture plates in a CO_2_ incubator for 24 h. Then, 3% paraformaldehyde was used to fix the cells. DAPI staining was then carried out. The cells’ nuclear morphologies were observed on a Leika DM 5000B fluorescence microscope produced in Wetzlar, Germany.

### 2.8. Statistics

The obtained values were presented as means ± standard deviation. In order to estimate the statistical significance of the data, a one-way ANOVA, post hoc comparison test and GraphPAD PRISM program were used. Variables * *p* < 0.05, ** *p* < 0.01, and *** *p* < 0.001 were accepted as significant.

## 3. Results

### 3.1. Preparation and Observation

**Figure 1:**
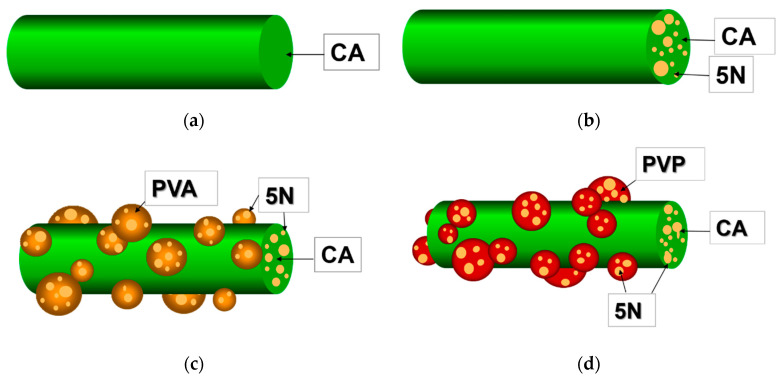
Schematic representation of: (**a**) CA fiber; (**b**) CA fiber loaded with 5N in the bulk (5N-*in*-CA); (**c**) CA fiber loaded with 5N in the bulk decorated with PVA,5N particles on the surface (PVA,5N-*on*-(5N-*in*-CA)); (**d**) CA fiber loaded with 5N in the bulk decorated with PVP,5N particles on the surface (PVP,5N-*on*-(5N-*in*-CA)).

In the present study, the following approaches were applied to fabricate innovative fibrous materials loaded with a biologically active substance—5N—with a diverse design: the one-step electrospinning of CA and 5N-*in*-CA fibers from a solution of CA or CA/5N (Figure 1a,b) and the electrospinning of a CA/5N solution with the simultaneous electrospraying of solution of PVA,5N or PVP,5N (Figure 1c,d).

**Figure 2:**
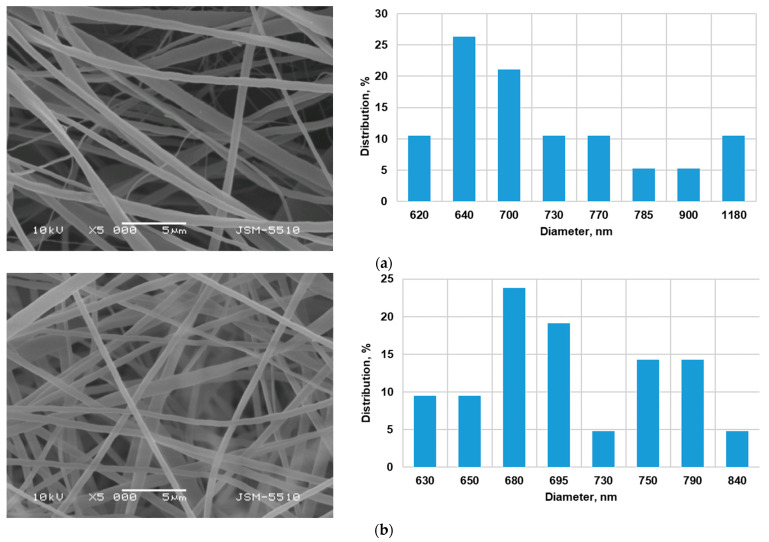

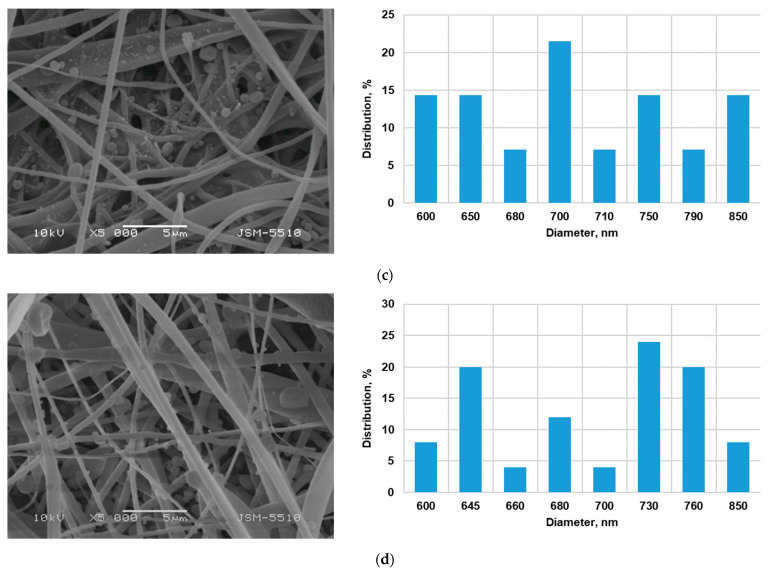
SEM micrographs and fiber diameter distributions of the fabricated materials: (**a**) CA fibers created via electrospinning; (**b**) 5N-*in*-CA fibers created via electrospinning; (**c**) PVP,5N-*on*-(5N-*in*-CA) created via electrospinning/electrospraying; (**d**) PVA,5N-*on*-(5N-*in*-CA) created via electrospinning/electrospraying. SEM magnification: ×5000.

Previously, we conducted a series of preliminary experiments to determine the optimal polymer concentration for the electrospinning of cellulose acetate [30]. It was found that defect-free fibers were obtained at a cellulose acetate concentration of 10 wt% in an acetone/water (80/20 *v*/*v*) solvent system. However, the influence of the incorporation of a biologically active substance (5N) on the fiber morphology and the biological activities of the hybrid materials also need to be studied. Figure 2 reveals the morphological structure and the distribution of the fiber diameter of the material fabricated via electrospinning/electrospraying materials. The electrospinning of solutions of CA and CA/5N (polymer concentration: 10 wt%) in an acetone/water solvent system (80/20 *v*/*v*) resulted in the fabrication of fibers without defects. The prepared fibers based on CA possessed a mean fiber diameter of 750 ± 230 nm (Figure 2a). The loading of 5N into the CA solutions led to an insignificant decrease in the mean fiber diameter of the prepared 5N-*in*-CA fibrous mats to 710 ± 210 nm (Figure 2b). The addition of 5N (10 wt%) to the PVP or PVA spinning solution (polymer concentration 2 wt%) for electrospraying in combination with the simultaneous electrospinning of a CA/5N solution resulted in the decoration of the fibers with nanoparticles and led to preparation of the PVP,5N-*on*-(5N-*in*-CA) and PVA,5N-*on*-(5N-*in*-CA) fibrous materials (Figure 2c,d). As can be seen from the presented SEM micrographs, the deposited PVP/5N and PVA/5N particles have spherical geometries. The mean particle sizes for the PVP,5N-*on*-(5N-*in*-CA) and PVA,5N-*on*-(5N-*in*-CA) mats were 408 ± 109 nm and 520 ± 110 nm, respectively.

As can be seen from the fluorescence micrographs in Figure S1b–d (Supplementary Material), all fabricated 5N-loaded mats exhibited intense fluorescence that was homogeneously distributed along the fibers. In addition, in the case of the PVP,5N-*on*-(5N-*in*-CA) and PVA,5N-*on*-(5N-*in*-CA) mats (Supplementary Material, Figure S1c,d) an intense fluorescence uniformly distributed in the particles deposited on the fiber surface was recorded as well. The detected phenomenon was most probably due to the presence of homogeneously distributed 5N with intrinsic fluorescence in the polymer fibers or in the polymer particles. In contrast, there was no fluorescence observed in the case of the CA mats (Supplementary Material, Figure S1a).

### 3.2. XRD Pattern

**Figure 3:**
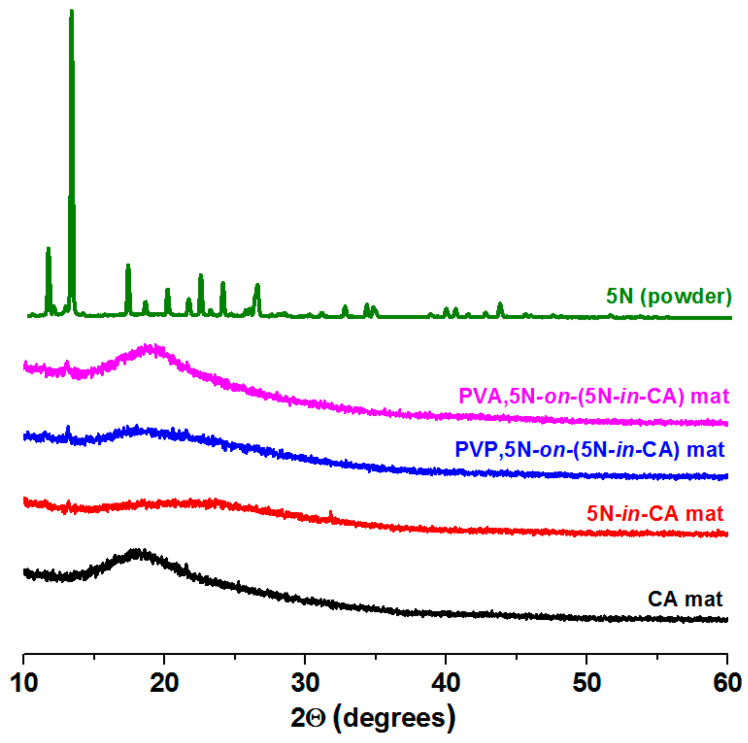
XRD patterns of 5N (powder) and fibrous materials of CA, 5N-*in*-CA, PVP,5N-*on*-(5N-*in*-CA) and PVA,5N-*on*-(5N-*in*-CA).

Figure 3 presents the XRD patterns of 5N (powder), the PVA,5N-*on*-(5N-*in*-CA) fibrous material, PVP,5N-*on*-(5N-*in*-CA), 5N-*in*-CA and the CA fibrous material recorded from 10 to 60°. Diffraction peaks were observed in the XRD pattern of 5N (powder), which shows that the initial substance in the powder form is in a highly crystalline state. The main crystallinity peaks were detected at 11.5°, 13.1°, 17.1°, 22.3°, 23,9° and 26.3°. XRD analyses showed that the CA electrospun material was in an amorphous form. In the spectra of all the 5N-containing fibrous mats, amorphous halos were detected as well. The absence of diffraction peaks for the crystalline phase of 5N is an indication that the biologically active substance loaded in the fibers and particles is in an amorphous state. This amorphization of the 5N is most probably due to the very fast process and solvent evaporation during the electrospinning/electrospraying, which do not allow for the crystallization of the drug.

### 3.3. FT-IR Studies

**Figure 4:**
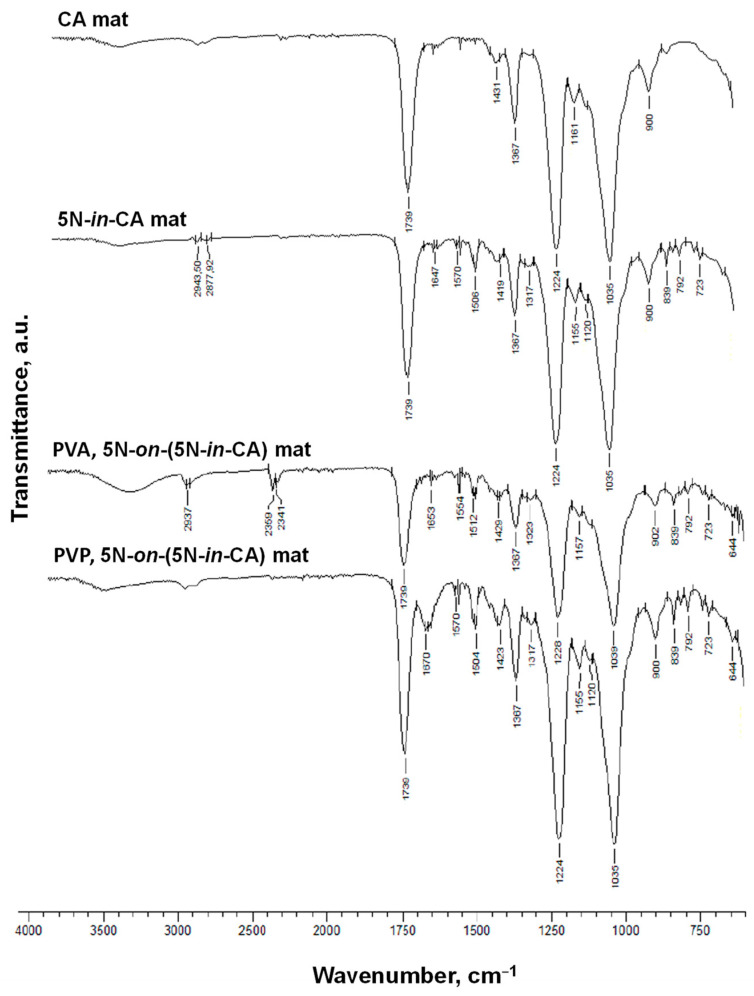
FTIR spectra of the CA, 5N-*in*-CA, PVA,5N-*on*-(5N-*in*-CA) and PVP,5N-*on*-(5N-*in*-CA) mats, recorded in the range of 4000 to 600 cm^−1^.

The CA and 5N-containing fibrous materials were analyzed using FTIR spectroscopy (Figure 4). The FTIR spectrum of the CA based fibers revealed bands characteristic of CA: 1739 cm^−1^ (C=O groups), 1367 and 1224 cm^−1^ (CH_3_ groups) and 1035 cm^−1^ (ether C–O–C groups) [29]. The spectrum of the 5N-*in*-CA sample (Figure 4) showed the presence of additional bands appearing at 1120 and 1506 cm^−1^ that are characteristic of the hydroxyquinoline derivative, revealing the loading of 5N in the samples. In the recorded spectra of PVA,5N-*on*-(5N-*in*-CA) and PVP,5N-*on*-(5N-*in*-CA), shown in Figure 4, peaks for the C=O functional groups at 1739 cm^−1^, for the the O-H functional groups at ~3500 cm^−1^, for the CH_3_ groups at 1367 cm^−1^ and 1224 cm^−1^, and for the ether C–O–C functional groups at 1035 cm^−1^, which are all characteristic of CA, were observed [37]. Moreover, bands of PVP at 1670 cm^−1^, 1317 cm^−1^ and 1423 cm^−1^, which are characteristic of C=O, C-N and CH stretching vibrations, were observed. In the spectrum of PVP,5N-*on*-(5N-*in*-CA) mat, characteristic bands for 5N were also detected (1504 and 1570 cm^−1^). In the PVA, 5N-*on*-(5N-*in*-CA) mat spectrum, characteristic PVA absorption bands at 1323, 1429, 2904 2937 cm^−1^ were observed. Characteristic bands for 5N were detected as well (1512 cm^−1^).

### 3.4. Water Contact Angle Measurements

**Figure 5:**
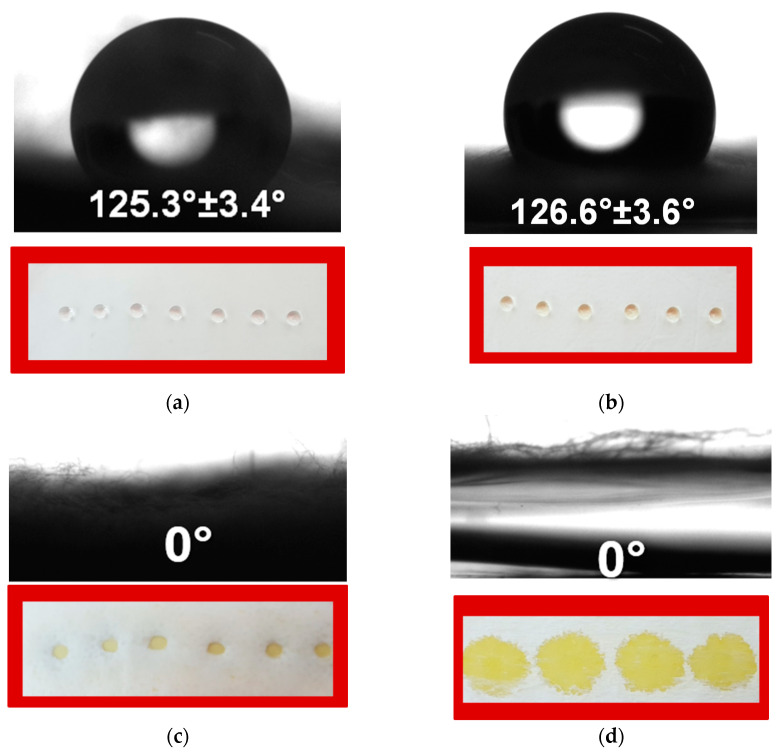
Digital photographs of deionized droplets of water placed on (**a**) CA, (**b**) 5N-*in*-CA, (**c**) PVP,5N-*on*-(5N-*in*-CA) and (**d**) PVA,5N-*on*-(5N-*in*-CA).

The surface hydrophobicity of the material affects the release profile of the drug from the fibrous mats and therefore plays an important role in bacterial and fungal adhesion and the manifestation of antioxidant and anticancer properties. As a result, it is critical to measure the contact angles of the fabricated electrospun samples that might come into contact with pathogenic species, cancer cells and normal cells. The contact angle was calculated by analyzing images of the droplets captured by the digital camera of the instrument. The obtained digital images of the water droplets as well as the contact angle values are shown in Figure 5. The results showed that the CA and 5N-in-CA mats (Figure 5a,b) were hydrophobic, with water contact angles of 125.3° ± 3.4° and 126.6° ± 3.6°, respectively. It was found that after the water droplet deposition, the droplets retained their spherical shape. The fibrous materials obtained via simultaneous electrospinning and electrospraying showed complete wetting, with contact angles of 0° (Figure 5c,d). This observation is most likely due to the presence of particles of the water-soluble polymers PVP and PVA, which led to the complete hydrophilization of the surface of the mats.

In terms of future applications of the prepared materials in the field of local cancer therapy, the hydrophilicity of the water-soluble-polymer-containing mats—PVP,5N-on-(5N-in-CA) and PVA,5N-on-(5N-in-CA)—is a crucial feature for enabling a drug’s rapid therapeutic effect. Furthermore, determining the water contact angle is critical for understanding the relationship between the composition of the mats and the prospective application of the mats as wound dressings. It is envisaged that PVP,5N-on-(5N-in-CA) and PVA,5N-on-(5N-in-CA) mats with contact angles of 0° will be promising as dressing materials to heal open wounds with greater levels of exudate leakage. In this case, an excessive amount of exudate will be absorbed by the dressing. The 5N-loaded mats will be also appropriate as dressings for the treatment of infected wounds.

### 3.5. Drug Release

**Figure 6:**
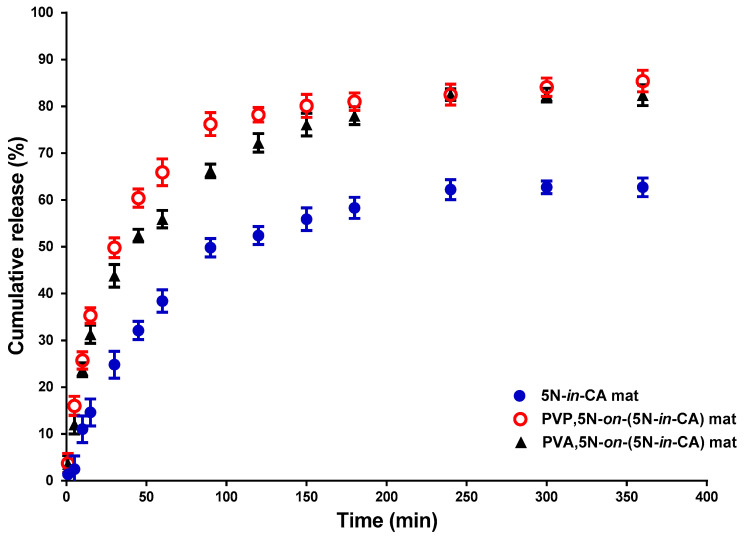
In vitro 5N release profiles from fibrous materials: 5N-*in*-CA, PVP,5N-*on*-(5N-*in*-CA) and PVA,5N-*on*-(5N-*in*-CA); acetate buffer, pH 5.5; 37 °C; ionic strength, 0.1.

Figure 6 shows the release of a drug from the electrospun fibrous materials prepared via electrospinning—5N-*in*-CA—or via electrospinning/electrospraying—PVP,5N-*on*-(5N-*in*-CA) and PVA,5N-*on*-(5N-*in*-CA). The 5N release was measured spectrophotometrically. Initially, the burst release of the drug from the fibers was determined. Then, a gradual mode of release was observed for all the types of mats, followed by a plateau within 350 min. The 5N-*in*-CA fibrous mat released the drug more slowly and released a lower amount (62.7%) compared to the PVP,5N-*on*-(5N-*in*-CA) and PVA,5N-*on*-(5N-*in*-CA) materials. The amounts of the 5N released in 175 min from the water-soluble-polymer-containing mats—PVP,5N-*on*-(5N-*in*-CA) and PVA,5N-*on*-(5N-*in*-CA)—were 85.4 ± 2.05% and 82.4 ± 1.89%, respectively. The observed variations in the release profile are most probably to the wettability of the different fibers. The contact angle values for the PVP,5N-*on*-(5N-*in*-CA) and PVA,5N-*on*-(5N-*in*-CA) were 0°, while for the 5N-*in*-CA, the measured value was 126.6°. The results from in vitro release experiments demonstrate that the release of 5N was facilitated by the presence of water-soluble PVP or PVA on the surface of the fibrous material. The acquired results are consistent with our previous reports on how the presence of a water-soluble polymer in the fiber or on its surface increases the drug release rate [17,18,29,30,33].

In the present work, the fabricated 5N-loaded fibrous materials demonstrated an initial stage of a fast release of 5N and a second stage of a gradual mode of release. The release behaviors detected may be advantageous for the prospective biomedical applications of the prepared materials as wound dressings or in local cancer therapy. An initial fast release of drug is desirable to inhibit bacteria growth in the infected wounds or to suppress cancer cell proliferation for cancer treatment. A gradual release is required to prevent the remaining bacterial or cancer cells from proliferating further. For this reason, the fabricated fibrous mats are suitable as wound dressings and for local cancer treatment.

### 3.6. Antioxidant Activity

**Figure 7:**
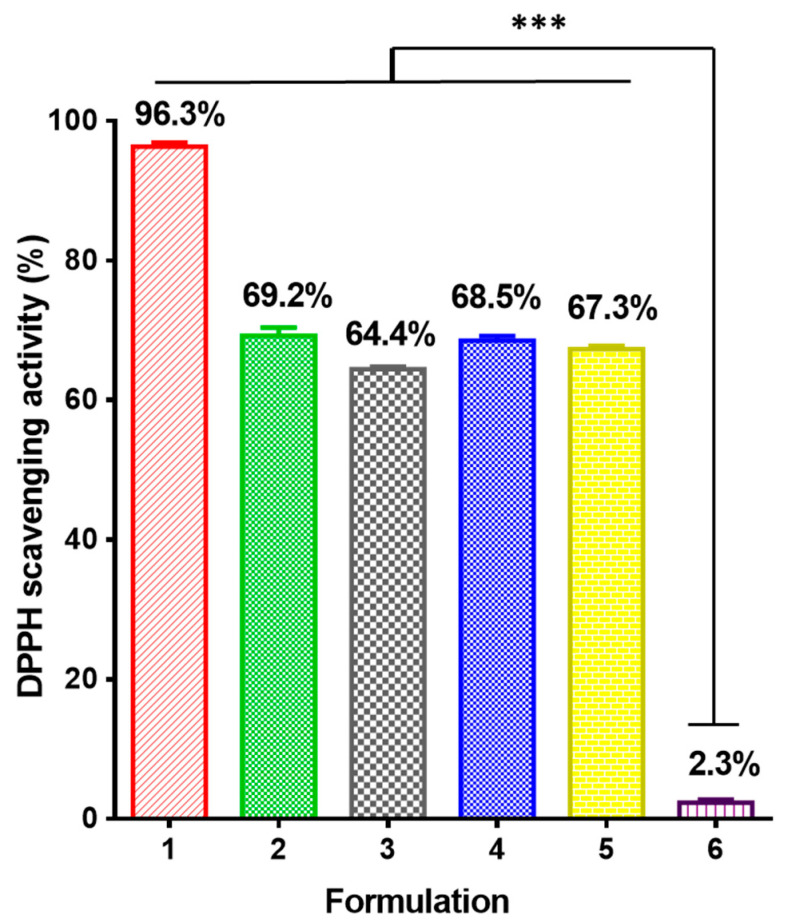
Comparison of antioxidant properties: 1) ascorbic acid solution in ethanol; 2) solution of 5N in ethanol; 3) 5N-in-CA mat, 4) PVP,5N-on-(5N-in-CA) mat; 5) PVA,5N-on-(5N-in-CA) mat; 6) CA mat. *** *p* < 0.001.

There are data in the literature that 5N possesses antioxidant activity [38,39]. However, the use of 8-hydroxquinoline derivatives as antioxidant agents is still scarce, and there are no data concerning the antioxidant properties of 5N-containing fibrous materials based on CA. In this study, the antioxidant activities of the free drug and the 5N-containing fibrous mats prepared via electrospinning/electrospraying were studied. The obtained results are presented in Figure 7. The antioxidant activities of the free 5N (2) and electrospun fibrous materials (3–6) were determined via DPPH testing, using ascorbic acid as a positive control (1). The DPPH radical scavenging activities of the CA-based electrospun materials were evaluated spectrophotometrically by monitoring the DPPH radical dot’s absorbance at λ 517 nm after the fibrous mats were incubated for 30 min at room temperature in the dark As can be seen from Figure 7, the DPPH scavenging of the CA fibrous mat had a minor impact on the DPPH solution (the DPPH radical’s absorbance lowered by 2.3%). Furthermore, after contact with CA fibers, the color of the DPPH solution was not drastically modified, and it preserved its violet color. On the other hand, the mats loaded with 5N exhibited good antioxidant activities (the DPPH• absorption statistically significantly decreased in the range of 64–69%, as can be observed in Figure 7 (3–5)). The color of the DPPH solution changed to yellow. The good antioxidant activities of the 5N-containing fibrous materials could be attributed to the metal-chelating property and electronic effects of 5N’s substituent groups. This result proved that the 5N incorporated into electrospun CA fibers or loaded in water-soluble polymer particles electrosprayed on the electrospun CA fibrous mat preserved its antioxidant activity and imparted antioxidant activities to the obtained novel fibrous mats.

### 3.7. Antibacterial and Antifungal Properties

**Figure 8:**
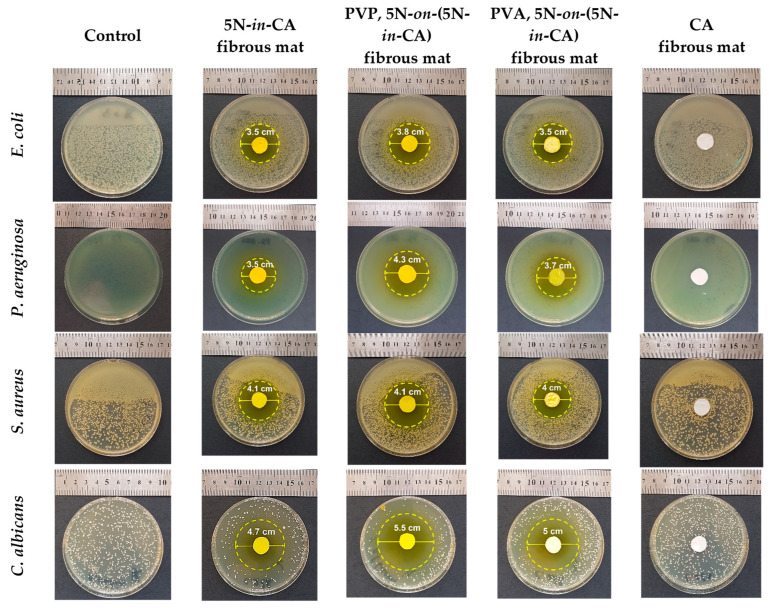
Digital photographs of the Petri dishes and zones of inhibition around the discs.

The antifungal and antibacterial properties of the fibrous materials were evaluated using microbiological assays against Gram-negative pathogenic bacteria—*E. coli* and *P. aeruginosa*— Gram-positive pathogenic bacteria—*S. aureaus—*and fungi—*C. albicans*. Digital images of the zones of inhibition are shown in Figure 8. A blank sample (control) showing the normal growth of the microorganisms is also presented. As expected, the CA mat did not possess any antibacterial properties and no zone of inhibition was observed. Well-distinguished sterile fields without bacterial and fungal growth were determined around the drug-containing CA mats. The presence of zones of inhibition indicates that the drug included in the mats preserves its antibacterial and antimycotic properties. The observation of zones of inhibition is evidence that the drug was incorporated. The addition of water-soluble polymers (PVA or PVP) into the CA fibers led to larger zones of inhibition compared to the 5N-in-CA mats. This result might be attributed to the easier drug diffusion caused by the improved hydrophilicity of the 5N-containing fibrous mats. Slightly larger sterile zones were detected on the PVP,5N-on-(5N-in-CA) mats compared to PVA,5N-on-(5N-in-CA) mats, which could be attributed to a greater release amount of 5N from the PVP-containing mats. The results showed that the fungi are more sensitive to 5N than the bacteria. The diameters of the inhibition zones around the 5N-in-CA, PVP,5N-on-(5N-in-CA) and PVA,5N-on-(5N-in-CA) mats against the fungi *C. albicans* were 4.7 cm, 5.5 cm and 5.0 cm, respectively. The antifungal mode of action of 5N is not well understood. According to some data from the literature, the antifungal activity mechanisms of the 8-hydroxyquinoline derivative manifest mainly because of damage to the cell wall, which compromises its integrity. Due to the loss of intracellular content and the subsequent disruption of the fungal membrane, 8-hydroxyquinoline and its related derivatives may target the ergosterol that forms transmembrane pores, changing the permeability of the membrane [40]. Moreover, it is important to note that around all 5N-containing mats, well-distinguished sterile zones with diameters of approximately 3.5 cm and greater centimeters were observed. This is evident proof that the 5N imparted a strong antibacterial and antifungal activity to the fibrous mats prepared via electrospinning or electrospinning/electrospraying.

### 3.8. In Vitro Cytotoxicity Assessment against Normal and Cancer Cell Lines

**Figure 9:**
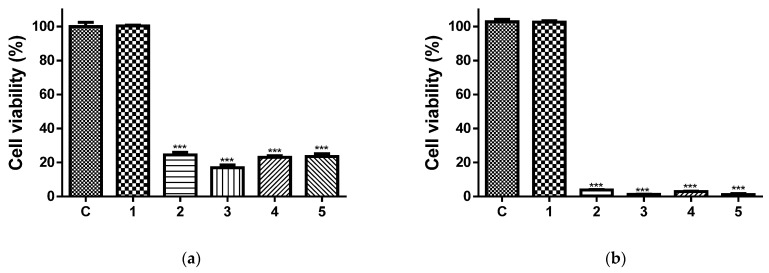

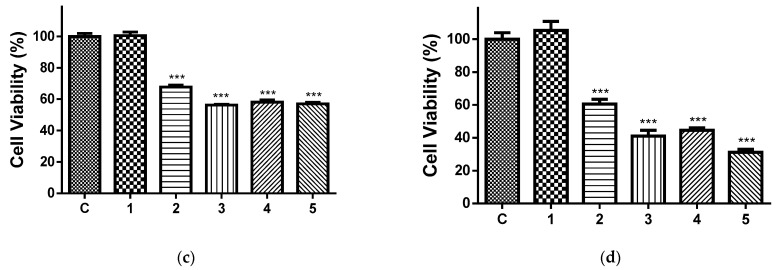
Cell viability assay of fibrous mats: HeLa cervical carcinoma (**a,b**) and fibroblast cells (**c**,**d**) after 24 h (**a**,**c**) and 48 h (**b**,**d**) in contact with the samples: C—HeLa or fibroblast cells (control); (1) fibrous mat based on CA; (2) 5N-in-CA fibrous mat; (3) PVP,5N-on-(5N-in-CA) fibrous mat, (4) PVA,5N-on-(5N-in-CA) fibrous mat and (5) free 5N (20 μM/L); *** *p* < 0.001.

Figure 9 presents the effects of the 24 h (Figure 9a) and 48 h (Figure 9b) contact of the HeLa cells with the free 5N and all types of electrospun non-woven materials on the cell viability. As can be seen, after 24 h and 48 h of incubation with the CA mat, the cell viabilities were 100.3 ± 1.5% and 102.6 ± 2.1%, respectively. Therefore, this biopolymer mat does not have any antiproliferative activity. In contrast, after 24 h of incubation with the free 5N (20 µM/L), the viability of the HeLa cells was significantly decreased. The percentage of the HeLa cells’ viability was 23.5 ± 2.2%. The results show that all the mats containing the 5N possessed high levels of antiproliferative activity. For the period of 24 h, the HeLa cells’ viabilities were reduced to 24.5 ± 1.7%, 16.8 ± 1.4% and 23.0 ± 1.5% for the 5N-in-CA, PVP,5N-on-(5N-in-CA) and PVA,5N-on-(5N-in-CA) fibrous mats, respectively. Furthermore, the PVP,5N-on-(5N-in-CA) and PVA,5N-on-(5N-in-CA) fibrous mats exhibited the highest levels of antiproliferative activity, which is probably due to the fact that 5N can be easily released from the more hydrophilic mats decorated with water-soluble particles, and the 5N can therefore easily manifest its anticancer activity. The MTT results after 48 h of incubation are shown in Figure 9b. It is obvious that the cell viability of the cancer cells was reduced further after contact with the 5N-containing formulations. The viability of the HeLa cells treated with the 5N-in-CA and PVA,5N-on-(5N-in-CA) fibrous mats was around 3%. Moreover, the viability of the cancer cells treated with free 5N and the PVP,5N-on-(5N-in-CA) fibrous mat was around 1%. These results showed that the 5N-containing fibrous materials exhibited a strong antiproliferative activity toward cancer cells.

Moreover, an in vitro cytotoxicity test against normal BALB/c 3T3 cells was also performed. The results presenting the effects of the materials upon contact with normal cells after 24 h and 48 h are shown in Figure 9c and Figure 9d, respectively. It is easily seen that the normal BALB/c 3T3 cells were less affected (Figure 9c,d) after contact with free 5N (20 µM/L) and 5N-containing fibrous mats. The percentages of cell viability were 67.7%, 56.3%, 58.1% and 57% after contact with the 5N-in-CA, PVP,5N-on-(5N-in-CA) and PVA,5N-on-(5N-in-CA) fibrous mats and free 5N, respectively. After 48 h, the cell viability continued to decrease. However, comparing the cell viability of the HeLa and BALB/c 3T3 cells, it is visible that the 5N-containing mats manifested strong antiproliferative effects toward the HeLa cancer cell line while retaining their lower levels of toxicity against normal BALB/c 3T3 cells.

### 3.9. Study of Cell Death by Staining with AO and EtBr and DAPI

**Figure 10:**
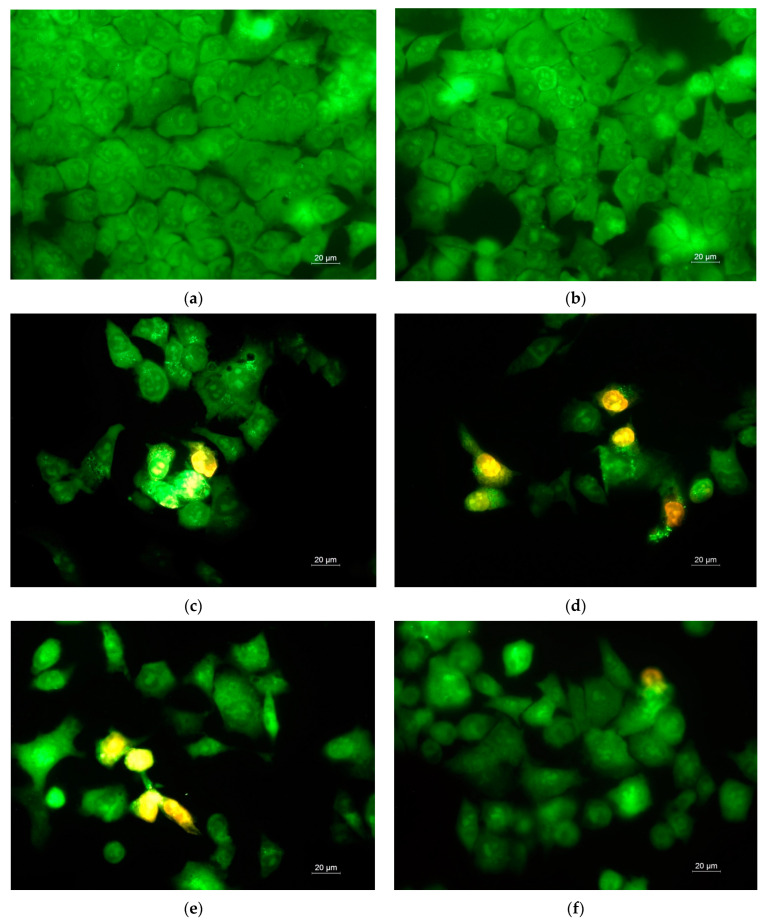
Fluorescence microscopy images of stained cancer cells after 24 h incubation with: (**a**) HeLa cells (control); (**b**) CA fibers; (**c**) 5N-*in*-CA fibers; (**d**) PVP,5N-*on*-*(*5N-*in*-CA) fibrous mat; (**e**) PVA,5N-*on*-*(*5N-*in*-CA) fibrous mat; (**f**) free 5N (20 μM/L). Live cells were green and dead cells were orange. Bar = 20 μm.

Analyses have been conducted to determine the extent to which apoptosis inhibits the growth of cancer and normal cells. Intravital double staining with fluorescent dyes (AO and EtBr) and DAPI was used to identify cell death. To study the morphological changes, HeLa and BALB/c 3T3 cells were cultured in the presence of the fabricated fibrous mats and free drug for 24 h and were then stained using fluorescent dyes. Fluorescence microscopy was used after staining to observe the cells. Supplementary Material, Figure S2, and Figure 10 present the fluorescent images of the HeLa cells double-stained with AO and EtBr and with DAPI, respectively. As can be seen from Figure 10, the control untreated HeLa carcinoma cells showed a homogenous, pale green nuclear fluorescence as well as bright yellow-green nucleoli. The human HeLa cells were numerous, and there were cells in a division phase as well (Figure 10 and Figure S2a). The morphologies of the cancer cells in contact with CA fibrous mat ware similar to those of the untreated cells (Figure 10b and Figure S2b). A slight thinning of the cells’ monolayers was observed. Significant reductions in cell numbers were detected when the HeLa cells were treated with 5N-containing mats and free 5N (Figure 10c–f and Figure S2c–f). Moreover, the cell shapes were changed, and significant modifications to the cells’ morphologies and their nuclei were detected. These changes were typical of apoptosis and included nuclear polymorphism, chromatin condensation, nuclei pyknosis and nuclei fragmentation, as well as the formation of apoptotic bodies (Figure 10c–f and Supplementary Material, Figure S2c–f). Thus, the obtained results demonstrate that fibrous materials loaded with 5N induced significant morphological changes in HeLa cancer cells, displaying strong levels of cytotoxicity toward this cancer cell line. The result of HeLa cell death, detected via AO/EtBr dual staining and DAPI staining, were in good agreement with the data obtained from the performed MTT assay.

**Figure 11:**
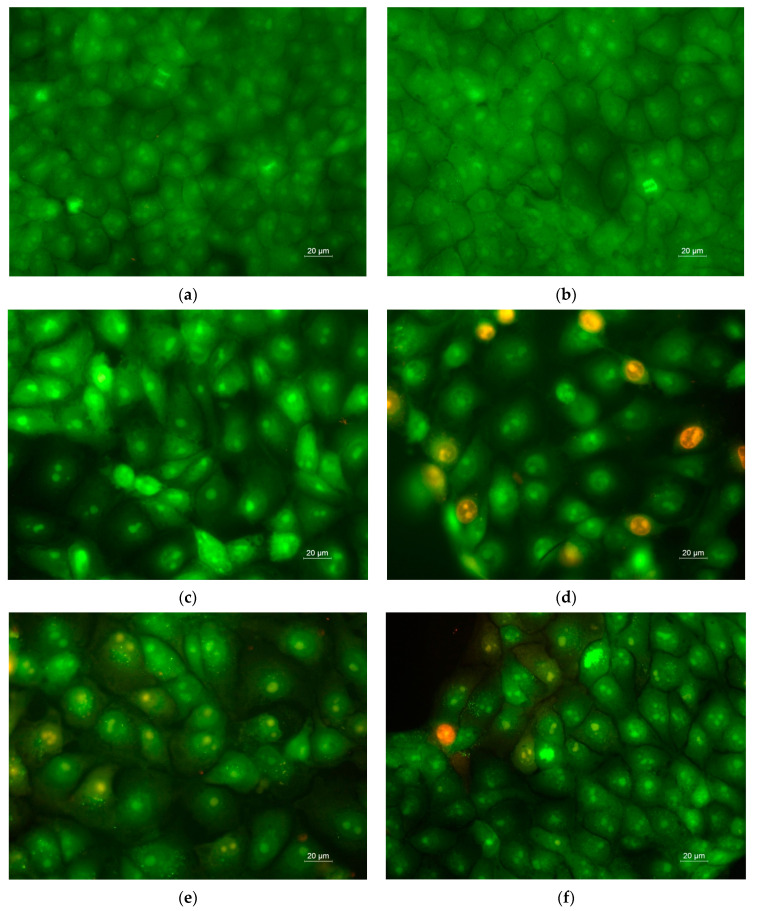
Fluorescence microscopy images of AO and EtBr double-stained BALB/c 3T3 mouse fibroblast cells after 24 h incubation with: (**a**) untreated BALB/c 3T3 cells; (**b**) CA fibrous mat; (**c**) 5N-*in*-CA fibrous mat; (**d**) PVP,5N-*on*-(5N-*in*-CA) fibrous mat; (**e**) PVA,5N-*on*-(5N-*in*-CA) fibrous mat; (**f**) free 5N (20 μM/L). Live cells were green and dead cells were orange. Bar = 20 μm.

The cell death of normal Balb/c 3T3 mouse fibroblasts after contact with the prepared novel fibrous materials was assessed as well. The fluorescent images, captured after double staining with AO, EtBr and DAPI, are presented in Figure 11 and Supplementary Material, Figure S3, respectively. It can be seen that when normal mouse fibroblasts were cultured in the presence of fibrous mats containing a water-soluble polymer loaded with 5N or free 5N, nuclear and cell morphological alterations, which are indicative of early and late apoptosis, were detected. (Figure 11d–f and Supplementary Material Figure S3d–f). However, the detected changes were noticeably reduced when the fibroblasts were treated with a 5N-containing fibrous mat and free drug compared to the cancer cells treated with the same formulations. The obtained results from studying the cell death via staining with AO and EtBr and DAPI revealed that the 5N-containing fibrous mats did not possess levels of toxicity against mouse fibroblast cells as strong as their levels of toxicity against HeLa cancer cells.

## 4. Conclusions

Electrospinning or simultaneous electrospinning and electrospraying were applied to obtain novel 5N-containing polymer fibrous materials with complex activities. The fabricated materials were morphologically, structurally and chemically characterized. XRD patterns showed that the 5N incorporated into the fibers or in PVP- or PVA-containing particles deposited on the fiber surface was amorphous. The distribution of the drug in the polymer fibers or in the polymer particles was homogeneous, as demonstrated by the fluorescence measurements carried out. The PVP,5N-*on*-(5N-*in*-CA) and PVA,5N-*on*-(5N-*in*-CA) fibrous mats evolved 5N at a greater rate when compared to the 5N-*in*-CA fibers. The 5N-containing mats showed considerable antibacterial and antifungal activities toward diverse pathogen types. Furthermore, the drug-containing mats prepared via electrospinning or through the conjunction of electrospinning and electrospraying displayed high levels of antiproliferative activity against human cervical HeLa cancer cells. The percentage of HeLa cell viability was reduced to around 3% after 48 h of contact with the 5N-in-CA, PVP,5N-on-(5N-in-CA) and PVA,5N-on-(5N-in-CA) mats. In contrast, these materials retained reduced levels of toxicity against normal fibroblast cells. The obtained results reveal that the innovative 5N-containing fibrous materials created in this study are suitable candidates for local cancer therapy as well as for dressings that promote wound healing.

## Data Availability

Data is contained within the article or supplementary material.

## Supplementary Materials

The following supporting information can be downloaded at: https://www.mdpi.com/article/10.3390/antiox12061243/s1, Figure S1: Fluorescence microscope images of fibrous materials: (a) CA, (b) 5N-*in*-CA, (c) PVP,5N-*on*-(5N-*in-*CA) and (d) PVA,5N-*on*-(5N-*in-*CA). Bar = 20 μm; Figure S2: Fluorescence microscope images of DAPI-stained HeLa cancer cells after treatment with: (a) untreated HeLa cells; (b) CA fibrous mat; (c) 5N-*in*-CA fibrous mat; (d) PVP,5N-*on*-(5N-*in*-CA) fibrous mat; (e) PVA,5N-*on*-(5N-*in*-CA) fibrous mat; (f) free 5N (20 μM/L). Bar = 20 μm; Figure S3: Fluorescence microscope images of DAPI stained Balb/c3T3 mouse embryo fibroblasts after treatment with: (a) untreated Balb/c3T3 cells; (b) CA fibrous mat; (c) 5N-*in*-CA fibrous mat; (d) PVP,5N-*on*-(5N-*in*-CA) fibrous mat; (e) PVA,5N-*on*-(5N-*in*-CA) fibrous mat; (f) free 5N (20 μM/L). Bar = 20 μm.

## References

[1] Reddy V.S., Tian Y., Zhang C., Ye Z., Roy K., Chinnappan A., Ramakrishna S., Liu W., Ghosh R. (2021). A Review on electrospun nanofibers based advanced applications: From health care to energy devices. Polymers.

[2] Islam M.S., Ang B.C., Andriyana A., Aff A.M. (2019). A review on fabrication of nanofbers via electrospinning and their applications. SN Appl. Sci..

[3] Fadil F., Affandi N.D.N., Misnon M.I., Bonnia N.N., Harun A.M., Alam M.K. (2021). Review on electrospun nanofiber-applied products. Polymers.

[4] Xu X., Ren S., Li L., Zhou Y., Peng W., Xu Y. (2021). Biodegradable engineered fiber scaffolds fabricated by electrospinning for periodontal tissue regeneration. J. Biomater. Appl..

[5] Hong J., Yeo M., Yang G.H., Kim G. (2019). Cell-electrospinning and its application for tissue engineering. Int. J. Mol. Sci..

[6] Farhaj S., Conway B.R., Ghori M.U. (2023). Nanofibres in drug delivery applications. Fibers.

[7] Gaydhane M.K., Sharma C.S., Majumdar S. (2023). Electrospun nanofibres in drug delivery: Advances in controlled release strategies. RSC Adv..

[8] Wsoo M.A., Shahir S., Mohd Bohari S.P., Mat Nayan N.H., Abd Razak S.I. (2020). A review on the properties of electrospun cellulose acetate and its application in drug delivery systems: A new perspective. Carbohydr. Res..

[9] Khoshnevisan K., Maleki H., Samadian H., Shahsavari S., Sarrafzadeh M.H., Larijani B., Dorkoosh F.A., Haghpanah V., Khorramizadeh M.R. (2018). Cellulose acetate electrospun nanofibers for drug delivery systems: Applications and recent advances. Carbohydr. Polym..

[10] Liang W., Hou J., Fang X., Bai F., Zhu T., Gao F., Wei C., Mo X., Lang M. (2018). Synthesis of cellulose diacetate based copolymer electrospun nanofibers for tissues scaffold. Appl. Surf. Sci..

[11] Puls J., Wilson S.A., Holter D. (2011). Degradation of cellulose acetate-based materials: A review. J. Polym. Environ..

[12] Suwantong O., Supaphol P. (2015). Handbook of Polymer Nanocomposites.

[13] Frey M.W. (2008). Electrospinning cellulose and cellulose derivatives. Polym. Rev..

[14] Aidana Y., Wang Y., Li J., Chang S., Wang K., Yu D.-G. (2022). Fast dissolution electrospun medicated nanofibers for effective delivery of poorly water-soluble drug. Curr. Drug Deliv..

[15] Maslakci N.N., Ulusoy S., Uygun E., Çevikbaş H., Oksuz L., Can H.K., Oksuz A.U. (2017). Ibuprofen and acetylsalicylic acid loaded electrospun PVP-dextran nanofiber mats for biomedical applications. Polym. Bull..

[16] Shibata T., Yoshimura N., Kobayashi A., Ito T., Hara K., Tahara K. (2022). Emulsion-electrospun polyvinyl alcohol nanofibers as a solid dispersion system to improve solubility and control the release of probucol, a poorly water-soluble drug. J. Drug Deliv. Sci. Technol..

[17] Tsekova P., Spasova M., Manolova N., Rashkov I., Markova N., Georgieva A., Toshkova R. (2018). Electrospun cellulose acetate membranes decorated with curcumin-PVP particles: Preparation, antibacterial and antitumor activities. J. Mater. Sci. Mater. Med..

[18] Yakub G., Toncheva A., Manolova N., Rashkov I., Danchev D., Kussovski V. (2016). Electrospun polylactide-based materials for curcumin release: Photostability, antimicrobial activity, and anticoagulant effect. J. Appl. Polym. Sci..

[19] Wang H., Chu C., Hao L., She Y., Li Y., Zhai L., Jiang S. (2015). Synthesis, antimicrobial, and release behaviors of tetracycline hydrochloride loaded poly(vInyl alcohol)/chitosan/ZrO_2_ nanofibers. J. Appl. Polym. Sci..

[20] Zhang J., Chen K., Ding C., Sun S., Zheng Y., Ding Q., Hong B., Liu W. (2022). Fabrication of chitosan/PVP/dihydroquercetin nanocomposite film for in vitro and in vivo evaluation of wound healing. Int. J. Biol. Macromol..

[21] Sun S., Hao M., Ding C., Zhang J., Ding Q., Zhang Y., Zhao Y., Liu W. (2022). SF/PVP nanofiber wound dressings loaded with phlorizin: Preparation, characterization, in vivo and in vitro evaluation. Colloids Surf. B.

[22] Viscusi G., Paolella G., Lamberti E., Caputo I., Gorrasi G. (2023). Quercetin-loaded polycaprolactone-polyvinylpyrrolidone electrospun membranes for health application: Design, characterization, modeling and cytotoxicity studies. Membranes.

[23] Gupta R., Luxami V., Paul K. (2021). Insights of 8-hydroxyquinolines: A novel target in medicinal chemistry. Bioorg. Chem..

[24] Al-Busafi S.N., Suliman F.E.O., Al-Alawi Z.R. (2014). 8-Hydroxyquinoline and its derivatives: Synthesis and applications. Res. Rev. J. Chem..

[25] Tanzer J.M., Slee A.M., Kamay B., Scheer E. (1978). Activity of three 8-hydroxyquinoline derivatives against in vitro dental plaque. Antimicrob. Agents Chemother..

[26] Spasova M., Manolova N., Markova N., Rashkov I. (2016). Superhydrophobic PVDF and PVDF-HFP nanofibrous mats with antibacterial and anti-biofouling properties. Appl. Surf. Sci..

[27] Kyuchyuk S., Paneva D., Manolova N., Rashkov I., Karashanova D., Markova N. (2022). Core/double-sheath composite fibers from poly(ethylene oxide), poly(L-lactide) and beeswax by single-spinneret electrospinning. Polymers.

[28] Spasova M., Manolova N., Paneva D., Rashkov I. (2004). Preparation of chitosan-containing nanofibres by electrospinning of chitosan/poly(ethylene oxide) blend solutions. e-Polymers.

[29] Spasova M., Manolova N., Rashkov I., Naydenov M. (2019). Electrospun 5-chloro-8-hydroxyquinoline-loaded cellulose acetate/polyethylene glycol antifungal membranes against Esca. Polymers.

[30] Spasova M., Manolova N., Rashkov I., Tsekova P., Georgieva A., Toshkova R., Markova N. (2021). Cellulose acetate-based electrospun materials with a variety of biological potentials: Antibacterial, antifungal and anticancer. Polymers.

[31] Ignatova M., Manolova N., Rashkov I., Markova N., Kukeva R., Stoyanova R., Georgieva A., Toshkova R. (2021). 8-Hydroxyquinoline-5-sulfonic acid-containing poly(vinyl alcohol)/chitosan electrospun materials and their Cu^2+^ and Fe^3+^ complexes: Preparation, antibacterial, antifungal and antitumor activities. Polymers.

[32] Ignatova M., Stoilova O., Manolova N., Markova N., Rashkov I. (2010). Electrospun mats from styrene/maleic anhydride copolymers: Modification with amines and assessment of antimicrobial activity. Macromol. Biosci..

[33] Ignatova M., Stoyanova N., Manolova N., Rashkov I., Kukeva R., Stoyanova R., Toshkova R., Georgieva A. (2020). Electrospun materials from polylactide and Schiff base derivative of Jeffamine ED^®^ and 8-hydroxyquinoline-2-carboxaldehyde and its complex with Cu^2+^: Preparation, antioxidant and antitumor activities. Mater. Sci. Eng. C.

[34] Ignatova M., Anastasova I., Manolova N., Rashkov I., Markova N., Kukeva R., Stoyanova R., Georgieva A., Toshkova R. (2022). Bio-based electrospun fibers from chitosan schiff base and polylactide and their Cu^2+^ and Fe^3+^ complexes: Preparation and antibacterial and anticancer activities. Polymers.

[35] Rasband W.S. ImageJ, US National Institutes of Health, Bethesda, MD (1997–2021). http://imagej.nih.gov/ij.

[36] Mosmann T. (1983). Rapid colorimetric assay for cellular growth and survival: Application to proliferation and cytotoxicity assays. J Immunol. Methods..

[37] Tsekova P., Spasova M., Manolova N., Markova N., Rashkov I. (2017). Electrospun curcumin-loaded cellulose acetate/polyvinylpyrrolidone fibrous materials with complex architecture and antibacterial activity. Mater. Sci. Eng. C.

[38] Cherdtrakulkiat R., Boonpangrak S., Sinthupoom N., Prachayasittikul S., Ruchirawat S., Prachayasittikul V. (2016). Derivatives (halogen, nitro and amino) of 8-hydroxyquinoline with highly potent antimicrobial and antioxidant activities. BB Rep..

[39] Faydy M., Djassinra T., Haida S., Rbaa M., Ounine K., Kribii A., Lakhrissi B. (2017). Synthesis and investigation of antibacterial and antioxidants properties of some new 5-subsituted-8-hydroxyquinoline derivatives. J. Mater. Environ. Sci..

[40] Pippi B., Lopes W., Reginatto P., Silva F., Joaquim A., Alves R., Silveira G., Vainstein M., Andrade S., Fuentefria A. (2019). New insights into the mechanism of antifungal action of 8-hydroxyquinolines. Saudi Pharm. J..

