# Supplementary material for: Innovative Fibrous Materials Loaded with 5-Nitro-8-hydroxyquinoline via Electrospinning/Electrospraying Demonstrate Antioxidant, Antimicrobial and Anticancer Activities
Source: Antioxidants (Basel). 2023 Jun 9;12(6):1243. doi: 10.3390/antiox12061243 (PMC10295047; doi:10.3390/antiox12061243)
Supplement: Supplementary file 1 [file antioxidants-12-01243-s001.zip › antioxidants-2420065-supplementary.pdf]

Supplementary Material

Innovative fibrous materials loaded with 5-nitro-8-hydroxyquinoline by electrospinning/electrospraying with antioxidant, antimicrobial and anticancer activities

Mariya Spasova [1,*], Nikoleta Stoyanova [1], Nasko Nachev [1], Milena Ignatova[1,*], Nevena Manolova [1], Iliya Rashkov [1], Ani Georgieva [2], Reneta Toshkova [2] and Nadya Markova[3]

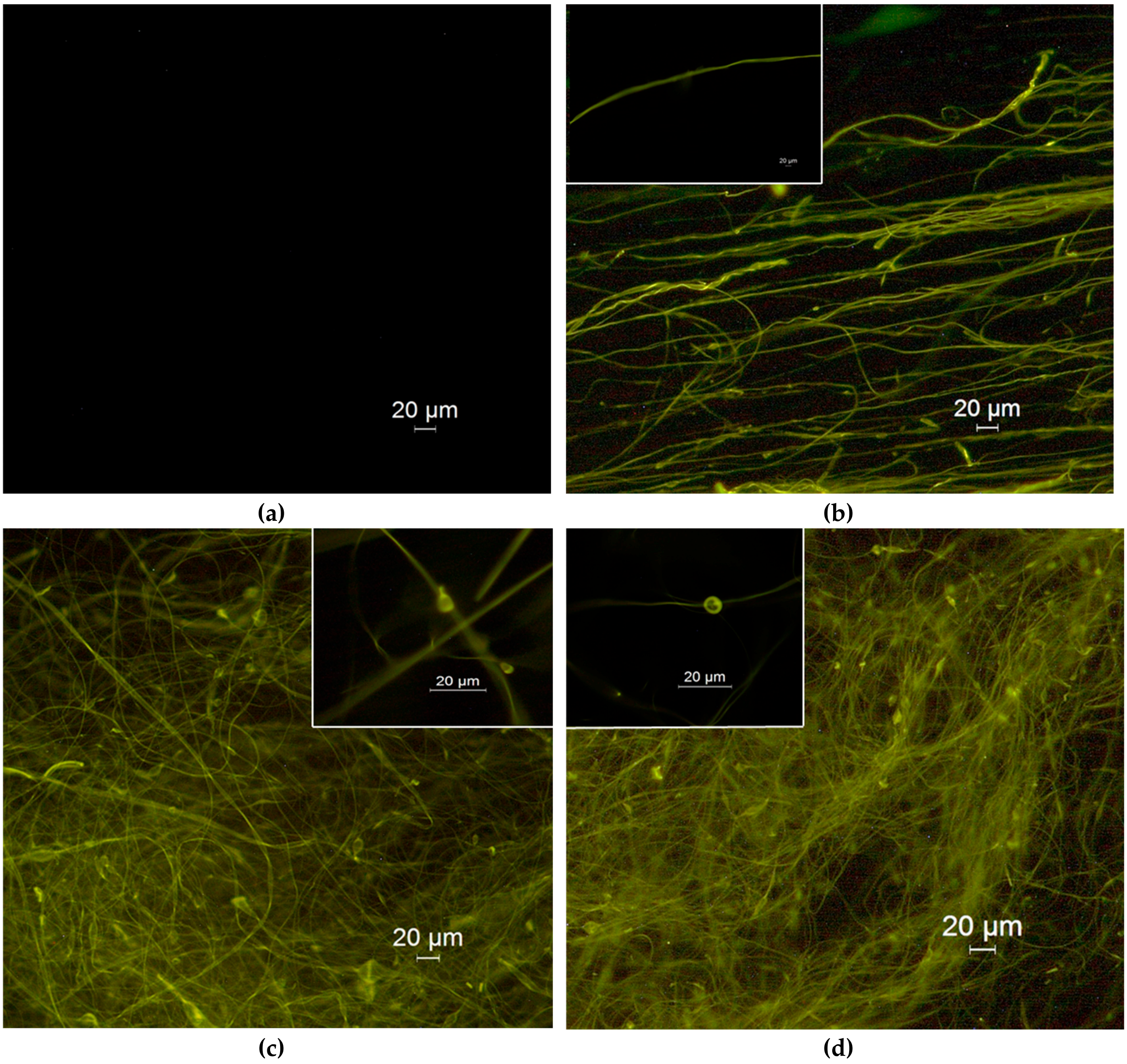

Figure S1. Fluorescence microscope images of fibrous materials: (a) CA, (b) 5N-*in*-CA, (c) PVP,5N-*on*-(5N-*in*-CA) and (d) PVA,5N-*on*-(5N-*in*-CA). Bar = 20 μm.

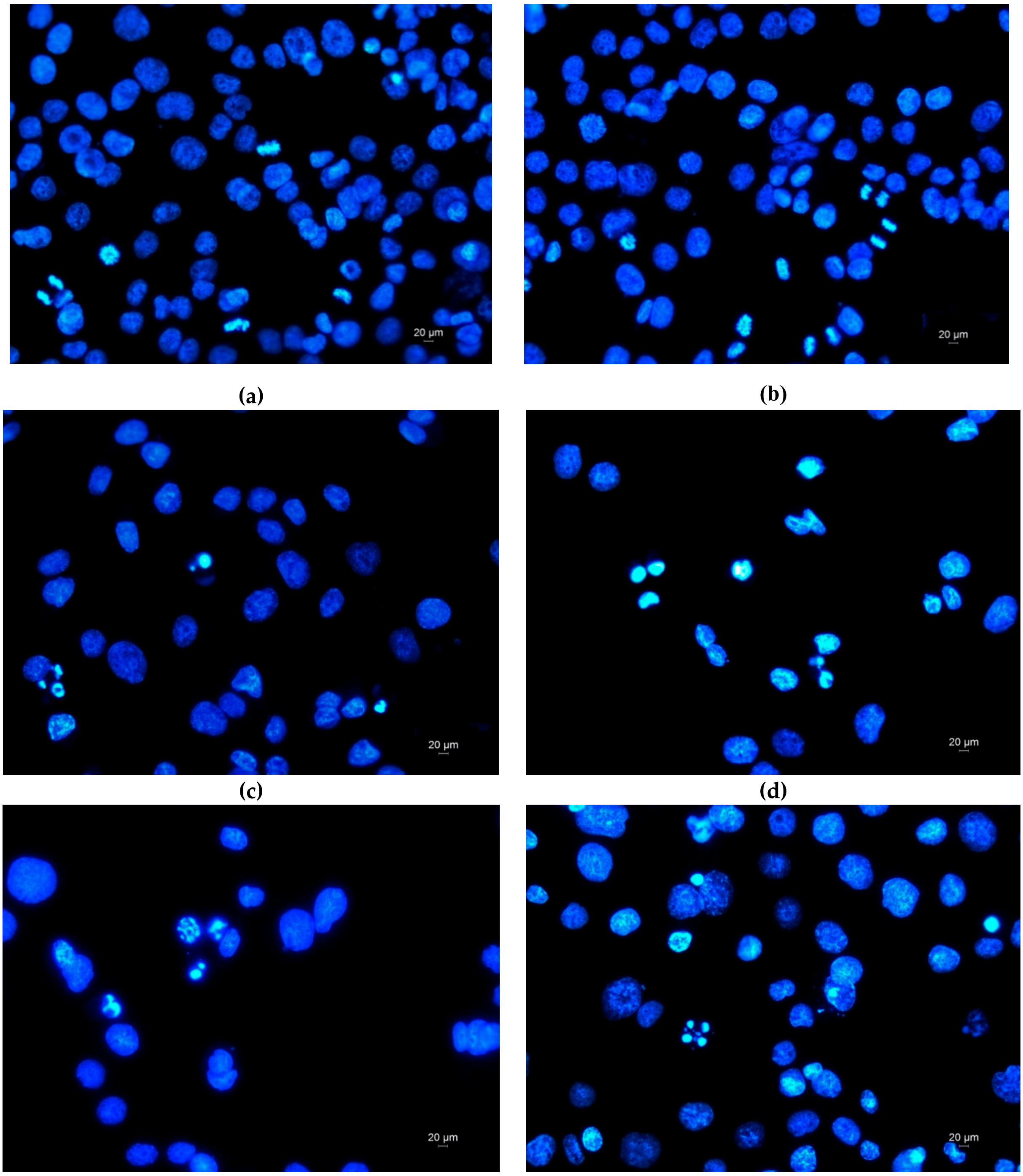

Figure S2. Fluorescence microscope images of DAPI stained HeLa cancer cells after treatment with: (a) Untreated HeLa cells; (b) CA fibrous mat; (c) 5N-*in*-CA fibrous mat; (d) PVP,5N-*on*-(5N-*in*-CA) fibrous mat; (e) PVA,5N-*on*-(5N-*in*-CA) fibrous mat; (f) free 5N (20 μM/L).Bar = 20 μm.

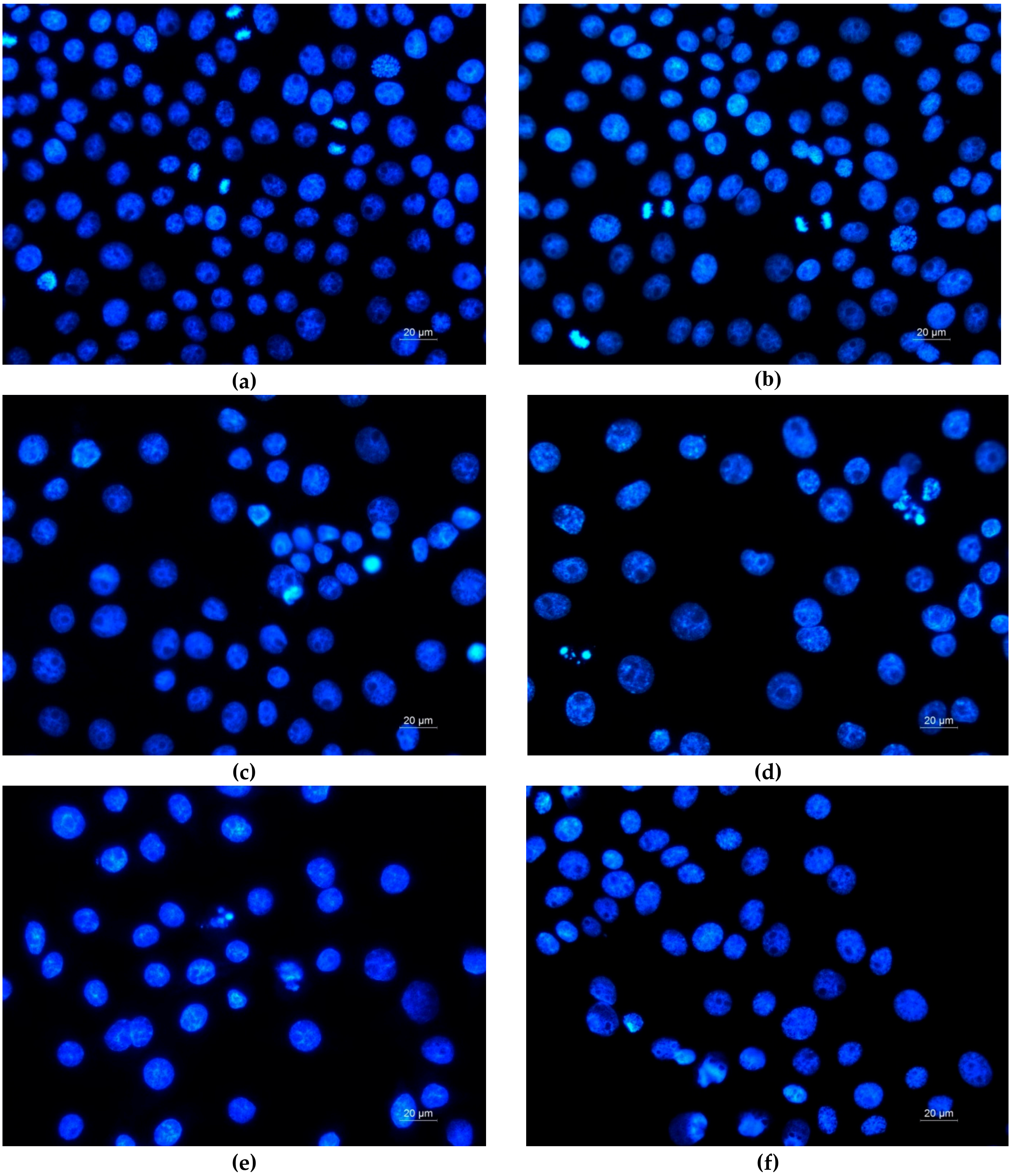

Figure S3. Fluorescence microscope images of DAPI stained Balb/c3T3 mouse embryo fibroblasts after treatment with: (a) Untreated Balb/c3T3 cells; (b) CA fibrous mat; (c) 5N-*in*-CA fibrous mat; (d) PVP,5N-*on*-(5N-*in*-CA) fibrous mat; (e) PVA,5N-*on*-(5N-*in*-CA) fibrous mat; (f) free 5N (20 μM/L).Bar = 20 μm.
